# Genetic, clinic and histopathologic characterization of *BRCA-*associated hereditary breast and ovarian cancer in southwestern Finland

**DOI:** 10.1038/s41598-022-10519-y

**Published:** 2022-04-25

**Authors:** Terhi Aino-Sofia Pallonen, Salla Maria Matleena Lempiäinen, Titta Kristiina Joutsiniemi, Riitta Irmeli Aaltonen, Pia Erika Pohjola, Minna Kristiina Kankuri-Tammilehto

**Affiliations:** 1 The Operational Division of Medicine, The Department of Clinical Genetics, Turku University Hospital, P.O.Box 52, 20521 Turku, Finland; 2 The Operational Division of Surgery and Cancer Diseases, The Department of Oncology and Radiotherapy, Turku University Hospital, P.O.Box 52, 20521 Turku, Finland; 3 The Operational Division of Obstetrics and Gynaecology, The Department of Gynaecologic Oncology, Turku University Hospital, P.O.Box 52, 20521 Turku, Finland; 4 The Operational Division of Surgery and Cancer Diseases, The Department of Plastic Surgery, Turku University Hospital, P.O.Box 52, 20521 Turku, Finland; 5 The Operational Division of Laboratory, The Department of Medical Genetics, Turku University Hospital, Turku, 52, 20521 Finland

**Keywords:** Cancer, Genetics

## Abstract

We have analyzed the histopathological, clinical, and genetic characteristics in hereditary breast and ovarian cancer patients of counselled families from 1996 up to today in the southwestern Finland population. In this study we analyzed the incidence of different *BRCA1* and *BRCA2* pathogenic variants (PV). 1211 families were evaluated, and the families were classified as 38 *BRCA1* families, 48 *BRCA2* families, 689 non-*BRCA* families and 436 other counselled families (criteria for genetic testing was not met). In those families, the study consisted of 44 *BRCA1* breast and/or ovarian cancer patients, 58 *BRCA2* cancer patients, 602 non-*BRCA* patients and 328 other counselled patients. Breast cancer mean onset was 4.6 years earlier in *BRCA1* carriers compared to *BRCA2* (p = 0.07, a trend) and ovarian cancer onset almost 11 years earlier in *BRCA1* families (p < 0.05). In *BRCA* families the onset of ovarian cancer was later than 40 years, and *BRCA2*-origin breast cancer was seen as late as 78 years. The *BRCA* PV (9%) increases the risk for same patient having both ovarian and breast cancer with a twofold risk when compared to non-*BRCA* group (4%) (95% CI p < 0.05). Triple-negativity in *BRCA1* (42%) carriers is approximately 2.6 times vs more common than in *BRCA2* carriers (16%) (p < 0.05). The risk ratio for bilateral breast cancer is approximately four times when compared *BRCA2* (17%) and other counselled patients’ group (4%) (p < 0.05). 27% southwestern *BRCA2*-families have a unique PV, and correspondingly 39% of *BRCA1*-families. The results of this analysis allow improved prediction of cancer risk in high-risk hereditary breast and ovarian families in southwestern Finland and improve long term follow-up programs. According to the result it could be justified to have the discussion about prophylactic salpingo-oophorectomy by the age of 40 years. The possibility of late breast cancer onset in *BRCA2* carriers supports the lifelong follow-up in *BRCA* carriers. Cancer onset is similar between *BRCA2* carries and non-*BRCA* high-risk families. This study evaluated mutation profile of *BRCA* in southwestern Finland. In this study genotype–phenotype correlation was not found

## Introduction

Breast cancer is the second most common cancer for females worldwide^1^. The risk for breast cancer is approximately 13% in Finland^2^ and approximately 5–10% of all breast cancers are inherited^3^. In Finland pathogenetic variants (PVs) that lead to a risk of 40% or higher for breast cancer are classified as high-risk variants^4^. Hereditary breast cancer susceptibility genes consist of high-risk variants and moderate-risk variants. It has been suggested that 25% of the hereditary breast cancer is due to *BRCA1* or *BRCA2* PV^5^. Published early studies in Finland 2000 and 2002 showed that *BRCA* PVs were associated with 20% of breast cancer families^6,7^, but lately the association has decreased as more patients are being tested due to widened gene test criteria, technological improvement in testing, and the refining of referral criteria and their easy discoverability online. In southwestern Finland the amount of *BRCA* PV in relation to all high-risk families so is 9.5% (unpublished observation). The ratio of *BRCA* PV in relation to all breast cancer patients varies geographically. In Finland proportion of *BRCA* breast cancer of all breast cancers is relatively low, the exact value is not known currently. In a Swedish study, the prevalence of *BRCA1* and *BRCA2* PVs was 1.8% of all unselected breast cancer patients^8,9^. Among breast cancer patients with cancer onset age under 40 years PV incidence has been shown to be higher than in other age groups: for example, in the United States Buys et al. observed *BRCA1* or *BRCA2* PV in about 8–14% of all young breast cancer patients^10^. In this study we investigated the onset of breast and ovarian cancer in different breast and ovarian cancer families according to family cancer risk type, result of *BRCA* test and type of *BRCA* PV. Additionally, we compared histopathologic characters in different risk groups.

The approximate risk of breast cancer is 65–79% with *BRCA1* PV and 61–77% for *BRCA2* PV^11^. The approximate risk of ovarian cancer is 40% for *BRCA1* PV and 20% in *BRCA2* PV^11,12^. Men with *BRCA2* PV have approximately a 6% risk of breast cancer, for men with *BRCA1* PV the risk is approximately 1%^13^. After 40 years of age *BRCA2* PV causes up to 5 times higher prostate cancer risk compared to men in general population^14^. 40–60 year old *BRCA1* carrier men’s cancer risk is twice that of men in general population^14^. Some genotype-fenotype correlation has been detected only in few PVs^15^. Currently, the knowledge about genotype–phenotype correlation is still not sufficient to use in individual risk assessment^16^. In our study we compared mutation profile to cancer onset.

Detecting families with *BRCA* PV is essential as it improves the cancer prognosis via follow-up and prophylactic surgery. Family’s females with *BRCA1* or *BRCA2* PV can participate in a breast screening. Most carriers have ovarian and fallopian tube removal that decreases ovarian cancer risk significantly and may halve the breast cancer risk^17–19^. It is also possible to organize gynecologic follow-up from 40 years onwards if patient does not want prophylactic bilateral salpingo-oophorectomy^20^. Skin-sparing mastectomy and breast reconstruction are also possible for patients with *BRCA* PV as mastectomy reduces breast cancer risk significantly^21,22^. For those *BRCA* PV patients who have had breast cancer it is possible to receive tamoxifen, to reduce risk of contralateral breast cancer, if prophylactic mastectomy is not done^23^. Usage of PARP inhibitors is possible in certain ovarian cancer patients including patients with *BRCA* PV^24^.

Currently gene testing is not done for all breast cancer patients as it has not been found cost-effective^25^, but there are studies investigating the cost-effectives of widespread *BRCA* screening^8,26^. *BRCA1* and *BRCA2* tumor analyses is done for all ovarian cancer patients.

## Methods

### Materials

A retrospective cohort study was made of all families who had had genetic counseling at the Department of Clinical Genetics in Turku University Hospital because of hereditary breast cancer suspect. Counseling has been held between 1996 and 2019. The counseled patients were given referrals from southwestern area of Finland and this area is named as “The expert responsibility area (ERVA) of the Tyks Turku University Hospital”. This analysis compares the onset of breast and ovarian cancer in *BRCA1, BRCA2*, non-*BRCA* and other counselled families. Genetic testing has been done for all patients except those in other counselled group, as they did not meet the criteria for genetic testing. We also compare the onset of breast and ovarian cancer in relation to different *BRCA* genes and to different *BRCA* pathogenic variants.

Families in our cohort are categorized to families with high breast cancer risk using modified familial high-risk criteria (Table 1). In our department genetic testing has been done after doctor’s evaluation based on family tree, patients medical record and genetic testing criteria. Genetic testing criteria has changed during the years, and we have followed the guidelines presented in Table 2 when evaluating possible benefits of genetic testing. Genetic testing in the family is always started from the family member who has had cancer, as then it is most likely to find the family PV. DNA is isolated from white blood cells in normal venous blood sample. If the family member has died, it is possible to isolate the DNA from one’s healthy tissue sample with relative’s approval.

**Table 1:** Family factors related to high risk for breast cancer in the study.

(I) One breast or ovarian cancer < 30 years old or
(II) Two breast or ovarian cancers and at least other one < 40 years old ﻿in 1st degree relatives or
(III) Three breast or ovarian cancers and at least one < 50 years old in 1st degree relatives or
(IV) Four breast or ovarian cancers at any age in 1st degree relatives
(V) One person have had both breast and ovarian cancer or
(VI) Male with breast cancer
(VII) Two first degree relatives with ovarian cancer even if there was no breast cancer in the family
(VIII) Five or more breast cancers in 1–3 degree relatives

**Table 2:** Current genetic testing criteria according to American Society of Clinical Oncology^25^.

1. The cancer of the person is suspected to be hereditary
2. The result of the genetic testing has a clear interpretation
3. Genetic testing provides at least one of next three benefits – Specifies the diagnosis or – Results to specific follow-up or – Provides information if patient benefits from a prophylactic surgery to reduce cancer risk

### *BRCA 1* and *BRCA2* gene test analysis

Since 1996 genetic testing methods have developed significantly. The whole gene sequencing is necessary as the mutation can locate in any part of the gene. Since 2011 both *BRCA* genes have been checked with Sanger sequencing. In addition, genes were tested with MLPA-reaction to detect deletions and duplications.

In 2017, next-generation sequencing (NGS) were used in 17% of breast and ovarian cancer panel. By 2019 all screening studies were done by NGS gene panel to obtain a family diagnosis. With gene panels it is possible to analyze multiple breast and ovarian cancer-associated gene mutations at once and it is faster than Sanger sequencing. For analysis, NGS libraries were prepared using BRCA Mastr Plus Dx kit (Agilent) and sequenced with Nextseq 500 sequencer (Ilumina). Bioinformatics analysis was performed with Sophia DDM (Sophia Genetics). Large genomic copy number variation was analysed with SALSA MLPA P002 and P045 probe kits for *BRCA1* and *BRCA2*, respectively. Fragment were analyzed with ABI 3500 xl Dx sequencer and GeneMarker software (Softgenetics).

Gene panel includes genes that are associated with increased breast cancer risk: *BRCA1*, *BRCA2*, *TP53*, *PTEN*, *STK11*, *CDH1*, *PALB2*, *CHEK2*, *ATM*, *FANCM.* Panel also includes genes that are associated especially with increased ovarian cancer risk (*BRIP1*, *RAD51C, RAD51D*) and genes associated with Lynch syndrome, which can increase the ovarian cancer risk (*EPCAM*, *MLH1*, *MSH2*, *MSH6*, *PMS2*)^4,25,27–30^. In this study we analyzed the incidence of *BRCA1* and *BRCA2* PV in cancer patients of counseled families.

### Variant nomenclature and classification

For variant classification, ACMG guidelines were used and variants were described according to HGVS nomenclature^29,30^. Pathogenicity predictions were made with Align GVGD, SIFT, Mutation Taster, PolyPhen-2 and CADD tools and Enigma, BIC, Clinvar, HGMD and GnomAD databases. Genbank reference sequences NM_007294.3 and NM_000059.3 were used for variant nomenclature.

### Statistical analysis

SAS Studio software version 3.8 (SAS Institute Inc., Cary, NC, USA) was used to perform statistical analyses. Sociodemographic and clinical variables were summarized using descriptive statistics, such as mean and standard deviation (SD) and frequencies and percentages. Dichotomous outcomes between different groups were reported using risk ratio (RR) with 95% confidence intervals (CI) and significance was analyzed using the Fischer’s exact test. Mean difference of cancer age was evaluated using Students’s T-Test, cancers with unknown age was discarded from the mean age test. All tests were two-sided and p-value less than 0.05 was considered to be statistically significant.

### Ethics approval and consent to participate

This study is hospital quality research, which has been authorized by Turku University Hospital and has valid ID. The study was not an experimental study. In the study analyzed data was from patients who had previously been treated at the hospital. Consent was obtained from all subjects or their legal guardians during treatment. All methods were carried out in accordance with relevant guidelines and regulations. As no new samples in this study were required a separate ethics board permit was not required. This is as guided by the ethics committee at Turku Clinical Research Center.

The Turku Clinical Research Center provides services in the field of health scientific research for researchers of the University of Turku and the Turku special responsibility area it also hosts the ethics committee.

## Results

1211 families were evaluated in southwestern Finland with clinical and family history that suggested hereditary breast and ovarian cancer. The families were classified as *BRCA1* families, *BRCA2* families, non-*BRCA* families*,* and other counselled families. The amount of cancer patients in these groups are shown in Table 3.

**Table 3:** Total number of cancer patients and families who were counseled in 1996–2020.

Counseled	BRCA1	BRCA2	Non-*BRCA*^1^	Others^1^
**Families**	38	48	689	436
**Cancer patients**	44	58	602	328
Breast cancer	23 (52%)	38^2^ (66%)	488^3^ (81%)	306 (93%)
Bilateral breast cancer	3 (7%)	10 (17%)	72 (12%)	13 (4%)
Ovarian cancer	13 (30%)	5 (9%)	19 (3%)	9 (3%)
Breast and ovarian cancer	4 (9%)	5 (9%)	23 (4%)	0 (0%)
Bilateral breast and ovarian cancer	1 (2%)	0 (0%)	0 (0%)	0 (0%)

^1^Non-*BRCA* and others group includes healthy relatives of a cancer patient, who is not from southwestern Finland and hence not included cancer patients.

^2^Includes 4 male breast cancer patients.

^3^Includes 28 male breast cancer patients.

Table 4 shows the amount of breast and ovarian cancer cases in the families and their details. Note that if the patient had a bilateral breast cancer, it was calculated as two breast cancer cases.

**Table 4:** Histopathologic and clinical characteristics of cancer cases.

Cancer cases	BRCA1	BRCA2	Non-*BRCA*	Others
**Breast cancers cases**	34	63	655	332
Breast cancer mean age at diagnosis and range	45.21 (27–67)	49.76 (29–83)	51.34 (23–84)	51.08 (30–87)
Triple-negative breast cancer cases	14 (42%)	10 (16%)	65 (10%)	45 (14%)
**Ovarian cancers cases**	18	10	42	9
Ovarian cancer mean age at diagnosis and range	50.71 (42–62)	61.50 (41–78)	58.16 (26–81)	53.00 (37–66)

**Figure 1:**
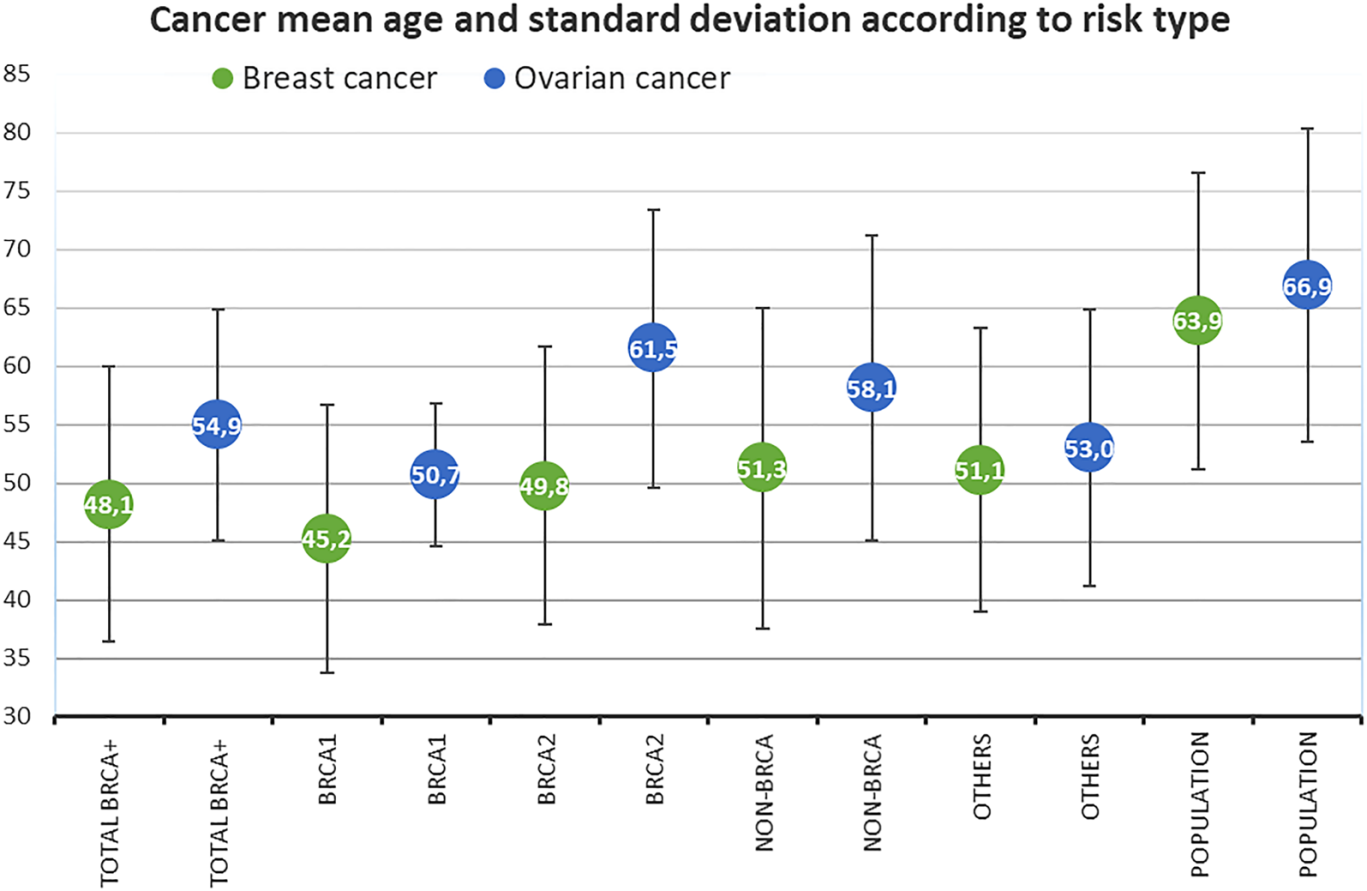
Cancer mean age and sample standard deviation in *BRCA*-families, non-*BRCA* families with high risk, other families and Finnish population.

Mean cancer age and sample standard deviation are shown in Fig. 1. The Finnish population data is added for reference and is based on Finnish Cancer Registry^2^.

Significance of breast cancer onset age was analyzed between groups by T-test. Table 5 shows significant or trending result of this analysis, non-significant results are not shown.

**Table 5:** Significant and trending differences in the mean onset of breast cancer.

Breast cancer	Mean onset age	Mean difference	p-value
*BRCA1* vs *BRCA2*	45.2 vs 49.8	− 4.6	0.071
*BRCA1* vs non-*BRCA*	45.2 vs 51.3	− 6.1	0.010
*BRCA1* vs others	45.2 vs 51.1	− 5.9	0.007

Significance of ovarian cancer onset age was analyzed between groups by T-test. Table 6 shows the significant or trending result of this analysis, non-significant results are not shown.

**Table 6:** Significant and trending differences in the mean onset of ovarian cancer.

Ovarian cancer	Mean onset age	Mean difference	p-value
*BRCA1* vs *BRCA2*	50.7 vs 61.5	− 10.8	0.004
*BRCA1* vs non-*BRCA*	50.7 vs 58.2	− 7.5	0.031

Triple-negativity was analyzed by calculating the risk ratio (RR) of triple-negative breast cancer patients between different groups with exact Fisher test. Significant and trending results are shown in Table 7, non-significant results are not shown.

**Table 7:** Significant and trending of tripe-negative breast cancer.

Triple-negative breast cancer	Risk ratio	95% CI	p-value
*BRCA1* vs *BRCA2*	2.6	1.3–5.2	0.012
*BRCA1* vs non-*BRCA*	4.1	2.6–6.6	< 0.001
*BRCA1* vs others	3.0	1.9–4.9	0.002

Bilateral breast cancer was analyzed by calculating the risk ratio (RR) of bilateral breast cancer patients between different groups with exact Fisher test. Significant and trending results are shown in Table 8, non-significant results are not shown.

**Table 8:** Significant and trending of bilateral breast cancer.

Bilateral breast cancer	Risk ratio	95% CI	p-value
*BRCA2* vs others	4.6	2.1–10.0	< 0.001

The risk ratio (RR) of a patient having ovarian and breast cancer (single or bilateral) was analyzed with exact Fisher test. Significant and trending results are shown in Table 9, non-significant results are not shown.

**Table 9:** Significant and trending of breast and ovarian cancer.

Breast and ovarian cancer	Risk ratio	95% CI	p-value
*BRCA1* vs non-*BRCA*	2.6	1.0–6.5	0.056

**Figure 2:**
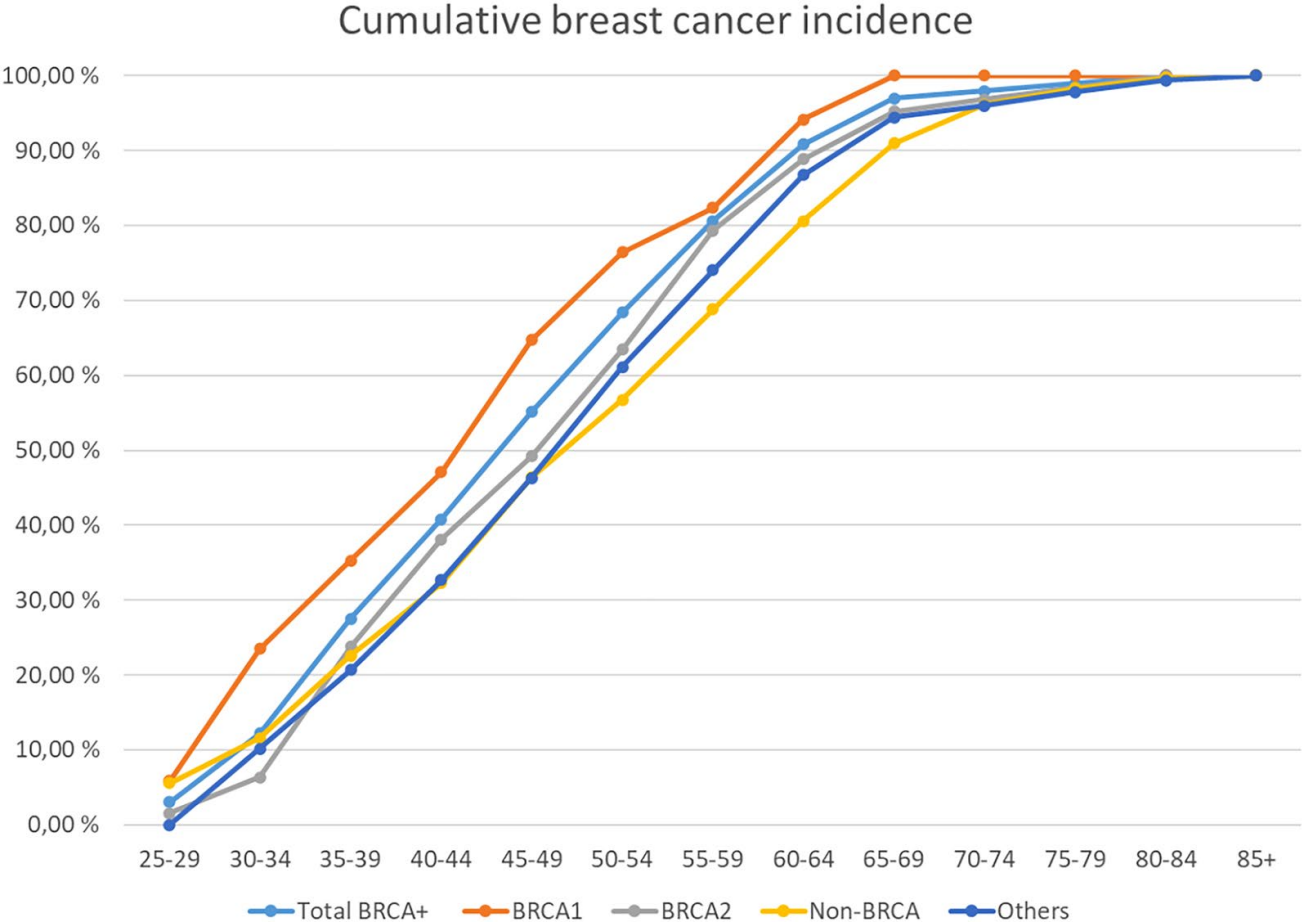
The onset age of breast cancer in *BRCA*-families, non-*BRCA* families with high risk and others. Exact values are marked with circles. Smoothing is used to make the curves more readable.

**Figure 3:**
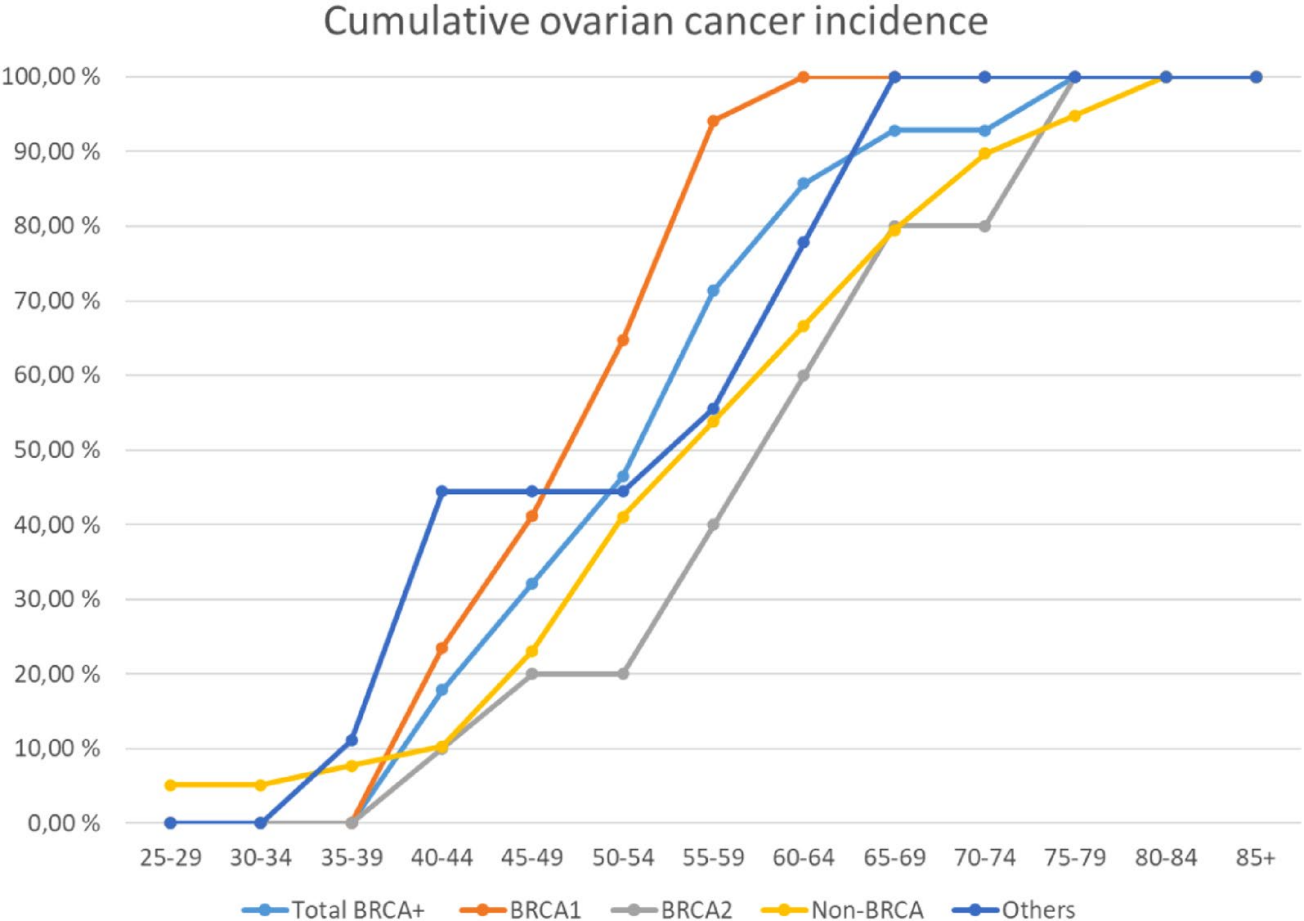
The onset of ovarian cancer in *BRCA*-families, non-*BRCA* families with high risk and others. Exact values are marked with circles. Smoothing is used to make the curves more readable.

Breast and ovarian cancer onset age was also evaluated with age brackets to compare their distribution. Due the difference in N values distribution instead of absolute values were used. Figure 2 shown the cumulative breast cancer cases as a function to age and Fig. 3 for ovarian cancer cases correspondingly.

There are several *BRCA* PVs. If the same PV appears in several families, it is considered a founder PV. The list of founder PV is in Table 10. 15 *BRCA1* families have a PV that does not appear in other families. 23 *BRCA1* families have a common PV. 13 *BRCA2* families have a unique PV. 35 *BRCA2* families a common PV.

**Table 10:** Most common and founder *BRCA1* and *BRCA2* pathogenic variants in southwestern Finland and their appearance in other countries.

Pathogenic variant	Families	Appearance
BRCA2 c.771_775delTCAAA; p.(Asn257Lysfs*17)	10	Founder mutation in Finland 4th common mutation in Caucasia^31,32^
BRCA2 c.9118-2A > G; p.(Val3040Metfs*20)	9	Founder mutation in Finland^33,34^
BRCA2 c.7480C > T; p.(Arg2494Ter)	7	Founder mutation in Finland^34^
BRCA2 c.3847_3848delGT; p.(Val1283Lysfs*2)	6	Founder mutation in Finland, also common in Scandinavian countries (Sweden, Norway, and Denmark). 5th common mutation in Caucasia^34–37^
BRCA1 c.3626delT; p.(Leu1209Ter)	5	Most common mutation Finland and in Northern Sweden^38,33^
BRCA1 c.4097-2A > G; p.(Gly1366fs*2)	4	Founder mutation in Finland^34,37^
BRCA1 c.3485delA; p.(Asp1162Valfs*48)	3	Founder mutation in Finland^34,37^
BRCA2 c.1286 T > G; (p.Leu429Ter)	3	Common in Finland^37^
BRCA1 c.4186-1787_ 4358-1668dup6081/6-KB DUP EX13	3	Common in Sweden and in English speaking countries^39^
BRCA1 c.5266dupC; p.(Gln1756Profs*74)	2	Founder mutation among Ashkenazi Jews. Most common founder mutation in Caucasia^37,40^
BRCA1 c.3756_3759delGTCT; p.(Ser1253Argfs*10)	2	Founder mutation in Russia and in French speaking Canada^41,42^
BRCA1 c.3607C > T; p.(Arg1203Ter)	2	Founder in Sweden^38^
BRCA1 c.4357 + 1G > A; p.(Arg1397Tyrfs*2)	2	–

Table 11 shows the three PV found in southwestern Finland and that are very rare in other parts of Finland.

**Table 11:** The pathogenic *BRCA1* and *BRCA2* variants in southwestern Finland, which are very rare in other parts of Finland and are rare also in southwestern Finland.

Gene	Pathogenic variant	Protein change
BRCA2	c.3530_3533delACAG	p.(Asp1177Alafs)
BRCA1	Whole gene deletion	
BRCA1	Exome 1–13 deletion	

**Figure 4:**
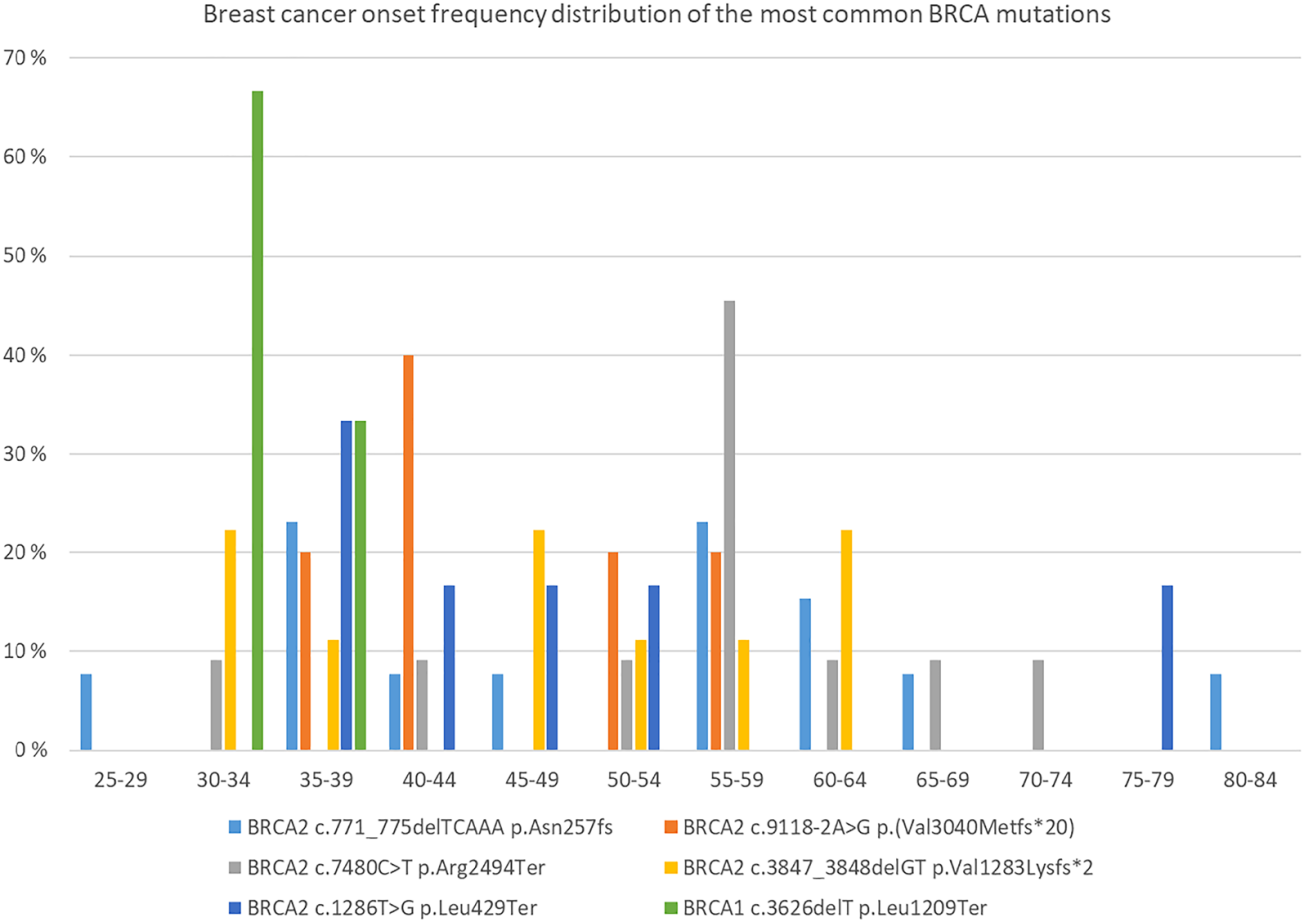
The onset frequency distribution of breast cancer by the most common different pathogenic variants.

The variants have a slightly different cancer onset age. Figure 4 shows this for the most common variants in southwestern Finland. There are no clusters in the breast cancer onset. The germline variants of BRCA1 and BRCA2 identified in this study are shown in Supplementary Table 1 and Table 2.

## Discussion

### Onset of breast and ovarian cancer in *BRCA1* and *BRCA2* families

In this study breast cancer onset was 4–6 years earlier for *BRCA1* patients compared to patients in all other groups. *BRCA1* compared to *BRCA2* result was only a trend, most likely due to the low number of breast cancer incidences. These observations are similar than reported in other studies^7,15^. Of interest is that in southwestern Finland breast cancer onset was similar between *BRCA2* families and in non-*BRCA* families and other counselled families.

In this study ovarian cancer onset was 8–11 years earlier for *BRCA1* patients compared to patients in *BRCA2* and non-*BRCA* group. Compared to others group the difference was not significant most likely due to low number of incidences. These observations are similar than reported earlier^15^.

Recommendations for follow-up programs are updated regularly^43^. In this study in *BRCA* families the onset of ovarian cancer is later than 40 years and breast cancer later than 26 years. Therefore, magnetic resonance imaging (MRI) screening for breast cancer from the age of 25 years is supported by our results. In non-*BRCA* group a very early onset of breast and ovarian cancer of less than 30 years’ was seen. This result may reflect that early breast cancer onset age is affected by polygenetic factors^44,45^, which are not well known currently. In our study, the risk of breast cancer decreases significantly after 70 years of age in *BRCA* patients but is still higher than in average population. The observation of our study supports the lifelong follow-up in *BRCA1* and *BRCA2* carriers as is the current recommendations^46^. MRI is recommended for *BRCA1* and *BRCA2* carriers^47^. After 70 years of age MRI can be replaced with mammography.

Risk-reducing prophylactic bilateral salpingo-oophorectomy is recommended for *BRCA* patients shortly after 40 years if the patient is willing for the surgery^48^. In the study of Kuchenbaecker et al.^11^ the incidence of *BRCA2* ovarian cancer is rising from the age of 50 years and *BRCA1* ovarian cancer over 10 years earlier^11^. It is opposite to the results of our study where the ovarian cancer onset for both *BRCA1* and *BRCA2* patients was soon after 40 years. According to the result it could be justified to have the discussion about prophylactic ovarian removal with a gynecologist by the age of 40 years.

### Other histopathologic and clinical features in *BRCA1* and *BRCA2*

The amount of triple-negativity represents 10–20% of invasive breast cancers in general population^49^. In our study triple-negativity is seen in 38% of *BRCA1* breast cancers. This is similar that has been seen in other studies^50^. Also, the ratio of triple-negativity between this study’s groups was in line with other studies^50^.

In all patients with breast cancer the cumulative incidence for contralateral breast cancer increases approximately 6% after 15 years^23,51^. Contralateral breast cancer risk is significantly higher in *BRCA* carries (about 39%)^23^. The 10-year risk of contralateral cancer is approximately 43% for *BRCA1* carriers and 35% for *BRCA2* carriers^52^. In our material in *BRCA2* carriers bilateral breast cancer was more common compared to *BRCA1* carriers, but this result was not significant.

This study also shows that the risk having both ovarian cancer and breast cancer is higher in *BRCA1* than in non-*BRCA* group (trend). This finding is in line with earlier studies, that have concluded that having both breast cancer and ovarian cancer raise the suspicion of *BRCA* PV.

### Type of pathogenic variants in southwestern Finland

More than 1800 pathogenic variants have been detected in both *BRCA1* and *BRCA2* genes^16^. This study is the first study, which investigates the mutation profile in southwestern Finland. So far there are 23 different PV types in *BRCA1* and 18 in *BRCA2* in counseled families. According to prior publications in the group of *BRCA2* families same PV appears more often in many families than in the group of *BRCA1* families^7,9^. We found that ten *BRCA2* families (21%) share the same PV c.771_775delTCAAA, which is very common in Finland, and 73% of all *BRCA2* families share a common PV. Of all *BRCA1* families 61% share a common PV. This observation is different to earlier studies in which in Finland 80% of *BRCA* PVs are common^7,9,33^. Our observation suggests that in southwestern Finland families more often have a unique mutation than in other parts of Finland.

We observed that all *BRCA2* PVs that were seen in more than a single families are also common in other part of Finland^4,6,9,33^. In Finland the large number of common PV in *BRCA* families is due to a strong founder effect. Finland is a geographically and culturally isolated country. A small population inhabited area that is nowadays known as Finland. The mutations of this population have enriched different Finnish regions over the years^32–34^. Spectrum of *BRCA1* founder PV is wider than *BRCA2* PV, where a small group of founder PVs are over presented in breast- and ovarian cancer families^32^. Due to the founder effect the most common founder PVs in Finland are not as common in Caucasian or European population, however there are some common PVs naturally^37^.

We also observed a common PV named 6-KB DUP EX13 (more specifically c.4186-1787_ 4358-1668dup6081), which is very rare in other part of Finland, but common in Sweden. To our best knowledge, this pathogenic variant has not been published in any other part of Finland. Common PV found in other parts of Finland, but that were not found in our study at Southwestern Finland were c.4327C > T, c.2684del2, c.5251C > T, c.1687C > T^32,34,37^. Large genomic alterations are uncommon in BRCA1 or BRCA2 gene in the Finnish population^53^.

### Type of pathogenic variant in association to onset of breast and ovarian cancer

In our study there were clear differences in the age of onset between different common PVs. For example, all cases of breast cancer for c.3626delT patients were before the age of 40. This information could be used to further improve when counselling is provided and when surveillance is started. However, the sample size was too small make statistical analysis of these differences.

Multiple breast cancer cluster regions (BCCR) and ovarian cancer cluster regions (OCCR) have been observed in *BRCA1* and *BRCA2* and are associated with relatively elevated breast cancer risk and lower ovarian cancer risk or inversely^15,54^. In our study’s for 56% (10/18) of all patients with *BRCA1* origin ovarian cancer the PV was located in the OCCR published in the study of Rebbeck^15^, whereas for *BRCA2* origin ovarian cancer no PV were located in the ovarian cluster region^15^.

Genotype–phenotype correlation is a topic for a follow-up study with greater family and patient amounts.

## Conclusion

In conclusion, more specific knowledge about different genetic prognostic factors allows us to evaluate the cancer risk and improve existing treatment guidelines. According to the result it could be justified to have the discussion about prophylactic salpingo-oophorectomy by the age of 40 years. The observation of our study supports the lifelong follow-up in *BRCA1* and *BRCA2* carriers as breast cancer can be diagnosed as late as approximately 80 years in *BRCA2* carriers. Onset of breast and ovarian cancer is similar between *BRCA2* carries and non-*BRCA* families. We observed that 39% of *BRCA1* and 27% of *BRCA2* family PVs were unique in Southwestern Finland. Genotype–phenotype correlation was not found in southwestern Finnish population in this study.

## Supplementary Information

Supplementary Tables.

## Data Availability

The data and materials are stored anonymously at the IT system of the Department of Clinical Genetics, Turku University Hospital, Turku, Finland.

## Abbreviations

NGS: Next generation sequencing
BCCR: Breast cancer cluster regions
BRCA: Breast cancer susceptibility gene
DNA: Deoxyribonucleic acid
PARP: Poly (adenosine diphosphate)-ribose polymerase
TNBC: Triple-negative breast cancer
