# Supplementary material for: Genetic, clinic and histopathologic characterization of BRCA-associated hereditary breast and ovarian cancer in southwestern Finland
Source: Sci Rep. 2022 Apr 25;12:6704. doi: 10.1038/s41598-022-10519-y (PMC9038668; doi:10.1038/s41598-022-10519-y)
Supplement: Supplementary file 1 — Supplementary Tables. [file 41598_2022_10519_MOESM1_ESM.docx]

## SUPPLEMENTARY INFORMATION

Supplementary Table 1. The germline pathogenic variants of *BRCA1* identified so far in southwestern Finland and the number of families who had it.

| **Variant** | **Number of families** |
| --- | --- |
| BRCA1 c.3626delT; p.(Leu1209Ter) | 5 |
| BRCA1 c.4097-2A>G; p.(Gly1366fs*2) | 4 |
| BRCA1 c.3485delA; p.(Asp1162Valfs*48) | 3 |
| BRCA1 c.4186-1787_ 4358-1668dup6081/ 6-KB DUP EX13 | 3 |
| BRCA1 c.5266dupC; p.(Gln1756Profs*74) | 2 |
| BRCA1 c.3756_3759delGTCT; p.(Ser1253Argfs*10) | 2 |
| BRCA1 c.3607C>T; p.(Arg1203Ter) | 2 |
| BRCA1 c.4357+1G>A; p.(Arg1397Tyrfs*2) | 2 |
| BRCA1 c.2273_2339dup; p.(Gln780Hisfs*4) | 1 |
| BRCA1 c.5074+1G>A; p.? | 1 |
| BRCA1 whole gene deletion | 1 |
| BRCA1 c.2685_2686delAA; p.(Pro897Lysfs*5) | 1 |
| BRCA1 c.1504_1508delTTAAA; p.(Leu502Alafs*2) | 1 |
| BRCA1 c.3706_3707delAA; p.(Asn1236Tyrfs*7) | 1 |
| BRCA1 del ex 1-13 | 1 |
| BRCA1 c.2475delC; p.(Asp825Glufs*21) | 1 |
| BRCA1 c.3145delT; p.(Ser1049Profs*13) | 1 |
| BRCA1 c.4035delA; p.(Glu1346Lysfs*20) | 1 |
| BRCA1 c.68_69delAG; p.(Glu23Valfs*17) | 1 |
| BRCA1 c.5147T>G; 5148A>T; 5149_5154del; p.(Leu1676Cys;Thr1677-Asn1678del) | 1 |
| BRCA1 promoter-ex 13 del | 1 |
| BRCA1 c.66_67AG; (p.Glu23fs) | 1 |
| BRCA1 c.4041_4042del; (p.Gly1348Asnfs*7) | 1 |

Supplementary Table 2. The germline pathogenic variants of *BRCA2* identified so far in southwestern Finland and the number of families who had it.

| **Variant** | **Number of families** |
| --- | --- |
| BRCA2 c.771_775delTCAAA; p.(Asn257Lysfs*17) | 10 |
| BRCA2 c.9118-2A>G; p.(Val3040Metfs*20) | 9 |
| BRCA2 c.7480C>T; p.(Arg2494Ter) | 7 |
| BRCA2 c.3847_3848delGT; p.(Val1283Lysfs*2) | 6 |
| BRCA2 c.1286T>G; p.(Leu429Ter) | 3 |
| BRCA2 c.4109T>A; p.(Leu1284Stop) | 1 |
| BRCA2 c.4169delT; p.(Leu1390Trpfs*20) | 1 |
| BRCA2 c.3283C>T; p.(Gln1095Ter) | 1 |
| BRCA2 c.2808_2811del; p.(Ala938Profs) | 1 |
| BRCA2 c.4936_4939del; p.(Glu1646fs) | 1 |
| BRCA2 c.8332-1G>T; p.? | 1 |
| BRCA2 c.3530_3533delACAG; p.(Asp1177Alafs) | 1 |
| BRCA2 c.6275_6276delTT; p.(Leu2092Profs*7) | 1 |
| BRCA2 c.2980_2984delGCAGG; p.(Ala994Thrfs*13) | 1 |
| BRCA2 c.3860dupA; p.(Asn1287Lysfs*2) | 1 |
| BRCA2 c.5621_5624delTTAA; p.(Ile1874Argfs*34) | 1 |
| BRCA2 c.751_754ACAG; p.(Asp252fs) | 1 |
| BRCA2 c.8327T>G; p.(Leu2776Ter) | 1 |
